# An alternate level of care plan: Co‐designing components of an intervention with patients, caregivers and providers to address delayed hospital discharge challenges

**DOI:** 10.1111/hex.13094

**Published:** 2020-06-30

**Authors:** Kerry Kuluski, Julia W Ho, Lauren Cadel, Sara Shearkhani, Charissa Levy, Michelle Marcinow, Allie Peckham, Jane Sandercock, Donald J Willison, Sara JT Guilcher

**Affiliations:** ^1^ Institute for Better Health Trillium Health Partners Mississauga ON Canada; ^2^ Institute of Health Policy, Management and Evaluation, Dalla Lana School of Public Health Toronto ON Canada; ^3^ Leslie Dan Faculty of Pharmacy University of Toronto Toronto ON Canada; ^4^ Rehabilitative Care Alliance / GTA Rehab Network / Toronto ABI Network Toronto ON Canada; ^5^ Edson College of Nursing and Health Innovation Arizona State University Tempe AZ USA

**Keywords:** Canada, co‐design, delayed hospital discharge, patient engagement, qualitative, transitions in care

## Abstract

**Objective:**

To engage with patients, caregivers and care providers to co‐design components of an intervention that aims to improve delayed hospital discharge experiences.

**Design:**

This is a qualitative study, which entailed working groups and co‐design sessions utilizing World Café and deliberative dialogue techniques to continually refine the intervention.

**Setting and Participants:**

Our team engaged with 61 participants (patients, caregivers and care providers) in urban and rural communities across Ontario, Canada. A 7‐member Patient and Caregiver Advisory Council participated in all stages of the research.

**Results:**

Key challenges experienced during a delayed discharge by patients, caregivers and care providers were poor communication and a lack of care services. Participants recommended a communication guide to support on‐going conversation between care providers, patients and caregivers. The guide included key topics to cover and questions to ask during initial and on‐going conversations to manage expectations and better understand the priorities and goals of patients and caregivers. Service recommendations included getting out of bed and dressed each day, addressing the psycho‐social needs of patients through tailored activities and having a storyboard at the bedside to facilitate on‐going engagement.

**Discussion and Conclusions:**

Our findings outline ways to meaningfully engage patients and caregivers during a delayed hospital discharge. Combining this with a minimal basket of services can potentially facilitate a better care experience and outcomes for patients, their care providers and families.

## 1 BACKGROUND

*‘Things just got different and I didn't know why’* explained Monika, a caregiver for her father who was waiting in hospital for a long‐term care bed. Monika's father eventually passed away, never making it to his next stage of care. The six other members of the Patient and Caregiver Advisory Council, with similar experiences, nodded in agreement, noting that hospital care had mostly stopped and they were confused as to what would happen next.

Alternate Level of Care (or ‘ALC’) is a construct used in Canada to refer to a delay in hospital discharge^1^; a common care quality challenge experienced across Canada^1^, ^2^, ^3^ and worldwide.^4^, ^5^, ^6^ In acute care, an ALC designation is typically given when a person has finished their medical treatment, has been cleared for discharge (usually by a physician) and is waiting for their next point of care^2^, ^7^ such as long‐term care (ie, nursing home), home with services, assisted living or rehabilitation. When these destinations are not available, a person is stuck waiting in hospital. Sixteen per cent of hospital beds in Ontario, Canada (the context of this research), are currently occupied by ‘ALC patients’, and this increases to one‐third of hospital beds in northern regions of the province.^8^ In Ontario, ALC numbers have steadily climbed over the past two decades despite attempts to address the issue.^9^ ALC is also costly to the health system, estimated at 2.5 million CAD each day in Ontario^10^ and 820 million pounds per year in the United Kingdom.^11^

In Canada, the Canada Health Act^12^ facilitates the provision of medically necessary hospital and physician care to all insured citizens based on need and not ability to pay.^13^ A key issue with ALC is that patients are at a stage where their needs are no longer considered to be *medically necessary* even though they are waiting in hospital. Thus, their care needs are considered low priority in favour of patients who require acute care. Since patients who are designated as ALC are often older adults with ongoing care needs, an absence or decrease in services can lead to functional decline,^2^, ^6^, ^14^, ^15^, ^16^ falls,^6^ risk of acquiring hospital‐borne infections,^17^ along with emotional stress^15^, ^18^ and feelings of uncertainty for both patients and caregivers (family members and friends who provide unpaid support).^15^, ^19^ ALC creates a ripple effect through the entire care pathway—starting in the emergency room, where patients may be stuck waiting for hours on stretchers in hallways (also referred to as ‘hallway medicine’)^20^ to cancelled surgeries and compromised access to post‐surgical rehabilitation and other treatments. ^6^, ^20^

That said, ALC is a symptom of a poorly functioning health‐care system,^20^ characterized by hospital process issues (delays in assessment or lack of consensus on next steps in care),^21^, ^22^, ^23^ post‐acute care capacity issues (lack of timely and suitable housing, rehabilitation and home care services)^5^, ^23^, ^24^, ^25^, ^26^, ^27^, ^28^, ^29^ or lack of long‐term care beds (particularly beds that are tailored to the linguistic, cultural and geographical preferences of patients and caregivers).^30^, ^31^, ^32^, ^33^ Simply put, the ALC problem represents a mismatch between available care services and the needs of patients and their caregivers. The few studies on patient and caregiver ALC experience report that patients and caregivers feel overwhelmed, confused, excluded from care conversations and concerned about the next steps in the patient's care.^15^ Providers, particularly discharge planners and social workers, feel stressed and mounting pressure trying to find a suitable care alternative.^6^ Strategies to address ALC challenges are required to improve patient, caregiver and care provider experiences. Addressing this issue is also important from a health system efficiency perspective.

While much can be done upstream and outside of hospital to adresss ALC challenges (e.g., developing community‐based infrastructure such as improved housing, enhanced home care services and paramedicine programmes to divert people from the emergency room), much can be done in hospital. Given the high risk of poor outcomes while waiting, designing a plan of care in hospital is important for patients with an ALC designation. Working with patients, caregivers and care providers to explore and design what better care could look like (ie, co‐design) is a necessary step towards improving experience. Care providers also need to be involved in the process, to ensure the feasibility of the design strategies and that they get the support they need in order to support patients and caregivers. Co‐designing strategies to address ALC challenges with patients, their caregivers and care providers is critical to ensure that care gaps are addressed and that services align with needs. Co‐design refers to working in partnership (typically with end users and providers) to design a service, programme or intervention that aims to improve care quality.^34^ There is emerging evidence to suggest that engaging with patients and caregivers as partners can lead to better care and outcomes.^35^, ^36^

The objective of our study was to work with patients, caregivers and care providers to co‐design components of an intervention to address commonly identified challenges during a delayed hospital discharge. Throughout this paper, we use the terms ALC and delayed hospital discharge interchangeably.

## 2 METHODS

This qualitative study entailed three phases: (a) the creation of a Patient and Caregiver Advisory Council, followed by (b) two working groups (one with patients/caregivers and the other with care providers) and (c) three co‐design sessions. The Advisory Council was formed in June 2018, and working groups and co‐design sessions took place between November 2018 and November 2019. The Advisory Council was comprised of people with ALC experience who actively guided and participated in all stages of the research. The goal of the working group with patients and caregivers was to learn about their experiences, map out their care journeys and identify key issues, from their perspectives, that need to be addressed to improve their experiences. For the provider working group, we summarized the issues identified by patients and caregivers as a starting place and captured providers’ perspectives on these issues and other pressing concerns. Finally, the co‐design sessions brought together patients, caregivers and care providers to develop strategies to address issues that were commonly identified in both the patient/caregiver and provider working groups. The co‐design sessions utilized principles from the World Café method^37^ (characterized by targeted small rotating group discussions where each conversation builds on the last) and deliberative dialogue^38^, ^39^ (determining the best course of action through discussion) which are two types of participatory research methods. World Café is intended to create a comfortable environment to exchange and build on ideas.^40^ In both the working groups and co‐design sessions, we aimed to create a comfortable atmosphere through smaller tables, rotating discussion and refreshments which is consistent with the principles of World Café.^40^ The deliberative dialogue principles of absorbing information, reflection, feedback and prioritizing were utilized in the co‐design sessions with the intention of identifying and reaching consensus on the intervention components. Each of these phases is explained below.

### 2.1 Advisory council

A recruitment flyer was distributed to hospitals based in Toronto, Ontario, Canada, to patients and caregivers who were currently (or had recently) experienced a delay in hospital discharge. The flyer was given to senior leaders from various Toronto‐based hospitals who connected with their care quality committees or patient relations departments to distribute the flyer. This resulted in six patient/caregiver partners who gave permission to their respective hospital lead for the lead author to contact them directly. All six individuals agreed to participate and attend a patient advisory kick‐off meeting to introduce themselves, share their experiences, confirm interest in joining the team and map out a work plan. At the meeting, all confirmed that they would like to stay involved and it was decided that additional patient perspectives were needed. With the help of a social worker at one of the local hospitals, a patient who was in hospital and who was designated as ALC was recruited and joined the Advisory Council. The Advisory Council was comprised of 7 members, both men and women, ranging from the age of forty to mid‐eighties, who were at various stages of their care journey (eg, current and bereaved caregivers as well as former and current patients). A chair was elected (caregiver member) and terms of reference created. Throughout the duration of the project, the tasks of the Council evolved from attending meetings, helping design/plan research materials and activities, and participating in the research itself as participants, to co‐facilitating co‐design sessions.

### 2.2 Working groups

The team held two working groups (akin to a focus group, but with more directed activities and guided discussions): one with patients and caregivers and then one with care providers. To be eligible to participate, patients had to be a current or previous ALC patient, and cognitively intact. Caregivers had to have experience caring for an ALC patient (with or without cognitive impairment). Care providers had to have experience caring for ALC patients. The patient/caregiver working group consisted of 11 participants (7 caregivers and 4 patients), 10 spoke English as their first language and another spoke Mandarin and was assisted by an interpreter. Other participants with sensory difficulties (speech and vision) were each supported by a community volunteer and member of the research team, respectively. All but one of the caregivers were providing care for family members, and the other was caring for a close friend. Four caregivers reported that their loved one had died during the delayed care transition. In the first half of the working group, participants articulated how they felt at each step of the care journey, which was determined by patients and caregivers as active hospital care, the stage when ‘things got different’ (ie, discharge delay), leaving hospital (for some) and back in the community waiting for placement (for some). Participants were handed activity sheets with a list of both positive and negative words to circle for each phase of the journey and had an opportunity to add new ones. The research team and Advisory Council designed the activity sheets, so the initial set of words were reflective of the Advisory Council experiences. The activity sheets served as an icebreaker at the beginning of the session. Filling out the activity sheets allowed participants to reflect on their experience and capture their thoughts and feelings in relation to stages of their care transition. The research team visually mapped their responses (positive and negative words onto butcher paper as a visual in the front of the room) and then used this as a tool to guide a more in‐depth discussion of their experience. Following this exercise, participants were asked to share the factors that shaped each phase of their journey and provide feedback on aspects that could improve.

The provider working group consisted of 12 participants; mostly point of care providers (social workers and discharge planners and some had backgrounds in nursing and occupational therapy). Some of the participants occupied a clinical manager role. The providers worked in various settings including acute hospital (4), post‐acute hospital (5) and community care sectors (3). Years in current practice ranged from 1 to 18.5 years. All providers spoke English as their primary language. Due to the size of the group, participants were broken into three smaller discussion tables to focus on key questions (informed by the findings of the patient and caregiver working group). All participants rotated through each of the three tables. The first table focused on communicating ALC (‘breaking the news’) with patients and caregivers; the second table focused on services for patients designated ALC; and the third table focused on the types of conflict experienced by care providers when a patient is ALC.

Extensive notes were taken by the research team with one member at each of the three tables. Notes from all sessions were read multiple times, line by line, by the lead author, an experienced qualitative researcher. Key ideas and emergent categories were outlined and then common ideas grouped together into core content areas which were then synthesized into core themes. The themes and corresponding notes were reviewed by members of the research team (specifically the members who took detailed notes) and Advisory Council to verify accuracy and suggest changes. For example, some of the descriptions of the themes were noted as confusing by the Advisory Council, so additional explanation and simplification of the terms were made. These core themes informed the content of the co‐design sessions described below.

### 2.3 Co‐design

Following the working groups, three co‐design sessions were held in Toronto, Sudbury and Mississauga, Ontario, representing geographically and culturally diverse regions of Canada's largest province. The same inclusion criteria from the working groups were applied. Participants were divided into one of three stations, which they subsequently rotated through. The commonly identified issues explored in the co‐design sessions, which were designed based on our working group findings were as follows: (a) communication (specifically, how to initiate the ALC conversation as well as the topics that should be covered in on‐going conversation) and (b) the services that should be provided during the ALC stage. Participants had an opportunity to first hear the findings, reflect on the findings, indicate whether they agreed or disagreed and provide recommendations for revisions. Following the Toronto co‐design session, key findings were synthesized and then presented in Sudbury to patients, caregivers and providers to garner their reactions and build on the findings. Finally, the synthesized findings of Toronto and Sudbury were presented in Mississauga to local caregivers and care providers for further consensus and refinement. In Toronto, the team engaged with 17 participants: 2 patients, 5 caregivers and 10 providers. In Sudbury, the team engaged with 19 participants: 10 patient/caregivers and 9 providers. In Mississauga, the team engaged with 17 participants: 10 caregivers and 7 providers. The providers mostly filled the role of discharge planning and were largely social workers by training, through some participants had other professional backgrounds including nursing, occupational therapy and medicine. The Advisory Council was engaged in all of these groups (as participants in the Toronto co‐design session and then as co‐facilitators in the Sudbury and Mississauga sessions).

## 3 RESULTS

### 3.1 Working groups

The top three words selected by the patient and caregiver participants across the care journey were as follows: stressed, frustrated and uninformed. All points of the care journey were mostly populated with negative feelings, though for those who experienced leaving the hospital (when the next care destination was finally determined) and plans were in place, positive emotions were more dominant. The following themes represent a synthesis of findings from the patient, caregiver and provider working groups.

#### 3.1.1 ALC is a confusing and variable term

Alternate Level of Care was described as a ‘Ministry term’ with no relevance to patients and caregivers. Patients and caregivers described varied experiences when receiving information from providers following an ALC designation, and it was generally a time of confusion and uncertainty. Providers explained that ALC was a term that was not 'one size fits all'. For example, when a patient moved from acute care to rehabilitation, it tended to be much more straightforward as they were ‘graduating to their next point of care’ and simply waiting for a bed to open up. In situations where patients were moving from hospital care to a new permanent home (such as long‐term care), it was much more complicated, particularly if people (providers, patients and caregivers) disagreed on the next care destination. Care providers described these two ALC scenarios as those who were either ‘*stuck*’ as they were waiting for a bed to open up or ‘*homeless in hospital*’ if there was no clear destination. This latter case also played out if a long‐term care home refused a patients’ application (often due to behavioural challenges).

#### 3.1.2 Communication with patients and caregivers is poor

Patients and caregivers wanted communication to be open and transparent. Caregivers suggested that communication tools and training to support providers (typically social workers/discharge planners who tended to be considered the point of care provider for ALC) were needed, especially when working with patients with dementia, language barriers or sensory impairments. Patients, caregivers and providers expressed the importance of having on‐going conversations with patients and caregivers to understand their preferences and goals of care. By unpacking patient and caregiver goals, providers could learn new ideas and get a better sense of what resources were needed to support a successful transition. Furthermore, having *early* conversations with patients and caregivers about issues such as housing barriers and other social factors was key to eliminate surprises and address issues that could ultimately lead to a delayed discharge. Providers articulated that they lacked the time to converse with patients and caregivers and provide on‐going psycho‐social care, as most of their time was spent looking for the next care destination.

Patients and caregivers received mixed messages from different members of the care team. Since the waiting period was highly uncertain, providers did not always feel they could adequately answer questions posed by patients and their caregivers. In these cases, it was perceived by patients and caregivers that care teams avoided conversations instead of acknowledging the uncertainty. The practice of holding *family meetings* tended to be reserved for situations characterized by conflict (such as when sibling caregivers disagreed on next steps). Team rounds were also a common practice for brainstorming how to support patients designated as ALC and explore care destinations, but typically excluded patients, caregivers and physicians. Including patients and caregivers along with physicians in team rounds was suggested as a way to get everyone on the same page and collectively solve problems. Finally, a lack of communication between care sectors (hospital and community) and care regions created additional confusion, particularly for discharge planners, given the different mixes of resources, eligibility requirements and wait lists.

#### 3.1.3 Caregivers feel isolated and want to be acknowledged

Caregivers felt isolated and excluded from the care team. Caregivers who were highly involved in the care of the patient wanted to have their insights acknowledged and valued by members of the clinical care team, as they held important patient information to support care planning. Even though caregivers themselves may need care and respite, the caregiver participants were primarily concerned with the patients’ welfare, not their own; however, some of the caregiver participants suggested that opportunities to connect with their peers (other caregivers having similar experiences) for advice and support would be helpful.

#### 3.1.4 There is a loss of basic services

Caregivers shared that the people to whom they cared for did not get out of bed often enough and were never dressed in regular clothes during the ALC period. They witnessed functional decline of their family members during the waiting period. Supports to prevent deconditioning, such as regular ambulation, were lacking. Other important and often overlooked services included chiropody (foot care), regular bathing, meal support, and laundry and recreation activities. These activities largely fell to caregivers to organize and execute. Co‐payments (similar to a long‐term care co‐payment) were required in some cases during the waiting period, but the level of care was described as low or non‐existent. Participants also wanted more time between bed turn‐over (the period of time when a patient leaves the hospital and another moves in) as both patients and caregivers felt rushed at discharge. They suggested that a separate room/lounge be available to get organized, review final logistics with the care team prior to transition to the next destination.

Services in hospital were identified to be sorely lacking, with an automatic decrease in care following an ALC designation. Some patients had access to light maintenance/rehabilitation (typically on a designated unit), while other patients did not have access to this care. Once designated ALC, the drop in rehabilitation was confusing for patients and caregivers as they witnessed other patients around them getting care. While some patients designated as ALC are ‘cohorted’ (placed in a similar unit) in some hospitals, others are mixed within units where other patients are getting treatment and rehabilitation. A patient participant from a cohorted unit with other ALC patients happened to report a better experience where she felt much more connected to her peer patients and was participating in group exercise among other activities available on the unit. In‐hospital service recommendations are summarized in Table 1 below.

**TABLE 1:** In‐hospital service recommendations

Domain	In‐hospital service recommendation
Activities of Daily Living and Hygiene	Getting out of bed and dressed once/day
	Bathing
	Foot care
	Regular ambulation
	Use of community day programmes
	Dental, ear and eye care
	Hairdressing
Instrumental Activities of Daily Living	Support with meals (support with eating as well as cooking groups)
	Laundry
	Finances (paperwork, applying for benefits)
	Managing upcoming prescriptions and appointments
Social and Mental Health Activation	My storyboard (one‐page patient backgrounder/visual) displayed by bedside/whiteboard.
	Pet therapy
	Religious activities/prayer group
	Spending time outdoors
	Connecting patients to peers on the unit
	Day passes to spend time in the community
	Recreation activities
	Music therapy
	Puzzles and crafts
	Books (hardcover and audio)
Logistics	A separate room/lounge for patients to make final arrangements/prepare for discharge to free up room for incoming patient

While the focus of much of the conversation was ‘what could be improved *within* hospital’, providers also emphasized a lack of programmes/services *outside* the hospital as a contributing factor to ALC. Providers underscored the importance of having options *other than* just long‐term care beds. Models of care that combine housing and care supports (such as assisted living and supportive housing) were valued but in short supply. There were also few resources to help adults and seniors deal with challenges related to the social determinants of health (low income, lack of social support, mental health and the intersection of these challenges). Providers were left to advocate for resources and search for the next care setting, leaving little time for on‐going, meaningful discussions with other members of the team and caregivers about next steps.

Providers spoke about a newly emerging option in the region of study called reactivation units (light transitional rehabilitation) situated within old hospitals re‐purposed specifically for ‘ALC patients’—serving as a conduit between hospital and their next care destination. Other promising community‐based programmes (including long‐ or short‐term housing with care services embedded, specifically tailored to the needs of people with chronic care needs) were referred to as ‘hidden gems’, were hard to find, in short supply, and were typically relatively small, grassroots organizations that were doing creative work to support people with various types of care needs. Furthermore, the lack of a centralized, ‘easy to navigate’ repository of available resources made these small but important services elusive.

Providers expressed frustration with the short‐term funding opportunities that came from the Ministry of Health. These ‘one time’ funding opportunities often coincided with an expectation that programme enhancements or new resources would be implemented quickly, and generally occurred during flu‐season, when spikes in hospitalizations were anticipated. Lack of sustained funding made it difficult to plan appropriately and think longer term.

### 3.2 Co‐design

#### 3.2.1 Station 1: How to initiate ALC conversations and manage expectations

To address the issues just described, patient, caregivers and care providers emphasized that it was important for point of care providers to know what was most important to every patient (not just those with, or at risk of, a delayed discharge) as well as any potential barriers for discharge. For patients that are designated as ALC, participants suggested that a point person be assigned to the patient/caregiver as their ‘go to person’ to provide on‐going updates and answer questions. It was important for patients and caregivers to know whom they could approach for questions and guidance, and that the ‘point person’ be connected to a broader team to access resources and avoid isolation. A specific list of questions that patients/caregivers and providers could ask throughout the ALC period was co‐designed in our sessions and is provided in Table 2.

**TABLE 2:** Question bank

	Questions	Reason for asking
For Providers to ask Patients and Caregivers (ie, family)	‘What is your understanding of how you're doing right now?’	To ‘level set’ across stakeholders and ensure that everyone's understanding of the situation is known
	‘What's most important to you?’	To ensure that the core concerns and priorities of patients and caregivers are captured (can help focus care planning)
	‘What are you most afraid of?’ or ‘What concerns you most?’	To get at the heart of what is most pressing for the patient and caregiver more directly.
	‘Are you satisfied with your progress?’	To capture how the patient and caregiver are feeling about their current state
	‘Were there any goals that you hoped to achieve?’	
	‘We are [an acute hospital] where we work together to [provide acute care and rehabilitation to patients like you]. We've offered what we are able to offer here, now we need to look at next steps…’	To manage expectations of patients and caregivers
	‘This part of your stay is over, you are now waiting for [next care setting] the [providers] won't come as often, therapy will decrease, etc. This is changing because [we are limited in the types of resources available]’.	
For Patients and Caregivers to ask Providers	‘Now that I’m here waiting for [next care setting] what will my day look like? Who will come see me? Will I still have some connection to the clinical team?’	To hold providers accountable and manage own expectations
	‘What are the next steps before discharge and how do we prepare?’	

#### 3.2.2 Station 2: Topics to cover in on‐going conversation

As outlined in Table 3, seven core topics were identified as key pieces of information that needed to be explored during the ALC stage with patients and caregivers. Topics related to patient and caregiver goals, preferences and fears; expectation management; and access to care. Once discharged, participants suggested that conversations between discharging and receiving care settings need to continue (particularly in the shorter term, such as shortly after the transition), including with the primary care doctor, geriatrician or home care team.

**TABLE 3:** Core categories and topics to address with ALC patients and their caregivers

Category	Topic
Assessing Patient and Caregiver Needs	Goals, priorities and concerns Caregiver availability and capacity
Managing Expectations	Reduction/change of care services Acknowledging uncertainty
Accessing Care	Physical barriers that limit discharge options Knowing what services are available, how to access them and who pays What medications are needed, what they are for and how they will be obtained

#### 3.2.3 Station 3: Services that should be provided during the ALC period

Participants recommended that the following four services be provided to all patients designated ALC. First, each patient should get out of bed and dress each day. From here, some mobilization could follow (a short walk or light exercises by the bedside or chair exercises if wheelchair bound). Second, attention to personal hygiene is required and should include bathing (more than once per week) as well as nail/foot care (if required). Third, use of whiteboards or storyboards was recommended (to be placed bedside) articulating who the patient was: such as their preferred name, personal interests, language spoken, favourite food and something interesting about their personal background. Fourth, attention to the social/mental health needs of the patient was suggested. Finding a specific approach to address this was difficult given the diversity of preferences. It was suggested that the care team, or designated provider or volunteer, work with the caregiver and patient to determine a socially/mentally stimulating activity within the means of the resources available (eg, books, crossword puzzles, connecting to religious services in hospital or with a peer patient). Given the general lack of resources and funding, participants started to explore ways in which care could be delivered, such as leveraging the volunteer sector, and incorporating student trainees (such as physiotherapy students doing hospital placements) to provide these supports.

## 4 DISCUSSION

Alternate Level of Care, the period of time where hospital care is complete but the next care setting needed is not readily known or available, is a confusing and stressful time for patients, their caregivers and care providers. Care typically decreases significantly without warning, and communication is often poor. To address this problem, our team collaborated with patients and caregivers to put together an Advisory Council for a multi‐stage project that explored how ALC experiences could be improved. The Advisory Council and research team conducted a number of working groups and co‐design sessions leveraging the World Café method and deliberative dialogue techniques to first understand issues related to ALC and then design components of a future intervention that would potentially address the identified issues. Patients, caregivers and providers participated from multiple care settings across Ontario, Canada. We discuss our findings under two core headings: *communicating uncertainty* and *ALC plan* and relate our findings to existing literature.

### 4.1 Communicating uncertainty

Poor communication in the health‐care system, generally (not just during care transitions), is a common problem expressed by many patients and their caregivers,^41^ typically those who have multiple chronic conditions,^42^ require significant health care and interact with various care providers across settings.^43^ Care transitions, in particular, are a heightened time of vulnerability for patients and their caregivers.^44^ In our research, we found that poor communication was particularly evident for patients, caregivers and care providers during a delayed care transition because the next step in care and associated timelines were often unclear. Figuring out *how* to have a conversation with patients and caregivers about what ALC means, as a first step, was critical. Social workers and discharge planners felt that this task largely fell to them, leading to feelings of isolation from the care team. Likewise, caregivers in our study felt ignored by staff, and had to ‘fight their way’ into conversations. Feeling isolated and ignored is a common theme found in other studies involving caregivers.^45^

Determining everyone's understanding of the situation was critical in order to avoid making assumptions and subsequent misunderstandings. Failing to assess the knowledge and understanding of a patient or caregiver can easily occur in health‐care settings, which have historically placed providers in the driver's seat when it comes to making decisions. Care providers may feel that they need to figure things out first, before engaging with patients and caregivers to plan next steps. While it is reasonable to take some time to gather information before speaking to patients and caregivers, if this period is too long, patient and caregivers can become confused and frustrated. In our study, we learned that caregivers and patients want to be involved in problem‐solving and be included as team members.

We found that communicating about the uncertainty itself (explaining to patients and caregivers that things would be different and uncertain as next steps were getting sorted out) was an important aspect of the conversation, yet something that tended to be overlooked. Understanding what *might* happen next (even if not concrete), feeling heard and understood are important to patients and caregivers.^46^ Opening up conversations with the question: ‘*What is your understanding of how you are doing right now?*’ was recommended by our participants. Following that, communicating the realities of the situation by acknowledging that care would not be the same and that things might be confusing for the next while was recommended but also noted as an awkward and vulnerable part of the conversation. While ideally, care would not be confusing and optimal levels of services would be provided, the reality of constrained health‐care resources and rationing, means it is important to be upfront with patients and caregivers about what is possible (while at the same time advocating for needed resources). Being honest can foster trust between patients, caregivers and the care team.

The types of questions co‐designed by patients, caregivers and care providers in our study, along with the suggested topics to guide on‐going conversation, can be used as a communication tool and guide for patients, caregivers and providers. Assigning a key point person from the care team, with a clear method of contact for patients and caregivers, can help them stay connected while waiting. It is also important for this point person to have access to a supportive team so they too, do not feel isolated. ‘ALC rounds’ where hospital and community providers come together to discuss challenging discharge scenarios was one promising approach, something which should be considered as a standard practice, albeit with patients and caregivers. The practice of *bedside rounding* is another promising practice adopted by a growing number of hospitals and could be explored for ALC situations. Hassmiller and Bilazarian^47^ found that when patients and caregivers were involved in rounds (discussions with the care team), their quality of care experience improved. Rounding at times when caregivers are present could help reduce isolation and help keep them informed, though this requires providers moving away from pre‐defined schedules and may not coincide with shift changes when these bedside rounds typically occur. Discharge summaries and tools (on paper or electronic) detailing pertinent pieces of patient information have been shown to support self‐management during and after care transitions, supporting a better patient and provider experience.^48^, ^49^, ^50^ Our communication tool outlines key topics and questions to support expectation management, the unpacking of patient and caregiver goals and capacity, and planning of next steps before the transition occurs. This communication tool is intended to be used throughout the duration of the ALC period.

Our findings are consistent with previous studies on shared decision making. Elwyn et al^51^ describe shared decision making as consisting of three components: introducing choice, describing options (typically with a patient decision support tool, which could be akin to our communication tool) and helping patients explore preferences. Elwyn et al note that ultimately, the decisions need to incorporate what matters most to patients and their caregivers.^51^ We argue that capturing the patient and caregivers’ *understanding* of their situation before *introducing choice* is important to ensure that everyone is on the same page. Joseph‐Williams et al^52^ observed patient‐clinician interactions in their research and found that shared decision making was much more complex, consisting of multiple stages including a preparatory phase (collecting necessary baseline information from patients including emotional state and personal circumstances from their charts or in‐person interactions), followed by on‐going interactive discussions, where choices are continually tailored based on personal circumstances, with some of these decisions distributed across additional members of the care team. Likewise, our findings highlight the importance of shared decision making across the broader team given that discharge planners currently feel isolated from allied health providers and physicians during the ALC period.

### 4.2 ALC plan

Participants acknowledged that some services should be provided in hospital, while patients are waiting to avoid risk of deconditioning and address low mood.Despite a standardized provincial definition and process^7^ for designating and reporting a patient as ALC, participants indicated there was no consistent approach to managing ALC, across hospitals. One commonality across hospitals was that an ALC designation typically coincided with a decrease in care. Patient and caregiver participants articulated how confusing it was to go from getting active treatment to almost nothing at all; hence, the expression, ‘*things got different and I didn't know why*’.

A list of services was recommended including getting out of bed and dressed once per day. This aligns with a programme launched in the United Kingdom, and recently adopted by Alberta Health Services in Canada called the *End PJ Paralysis initiative*.^53^ This initiative entails encouraging patients to do a combination of activities to keep them moving which could include getting up, out of bed and dressed, and doing mobility activities. These activities not only help to prevent physical deconditioning but facilitate a sense of personal dignity. For example, after the 70‐day PJ Paralysis initiative was implemented in the trauma and orthopaedic units at Nottingham University in the United Kingdom, there was a 37% reduction in falls, 56% reduction in pressure ulcers and 80% reduction in patient complaints.^53^ In addition to getting out of bed and dressed, attention to hygiene (bathing and foot care) was recommended by our participants.

Exploring ways to help patients feel socially connected (knowing their interests and exploring existing resources to meeting that need) and getting to know the patient through a storyboard (a poster by the bedside with answers to personal questions) were also prioritized components of the care plan and an important mechanism for engagement. In an environment of constrained resources, opportunities to leverage the volunteer sector and trainees (nursing, physiotherapy, occupational therapy and social work), with oversight from a member of the care team, will be an important consideration in further co‐design work. The value of volunteers (from both a patient experience and cost perspective) in the hospital sector has been demonstrated for a range of programmes including meals support,^54^ socialization and palliative/end‐of‐life care.^55^, ^56^ The role of volunteers in helping older patients mobilize in hospital is understudied according to a recent systematic review, though reports from a few studies suggest enhanced patient and provider satisfaction.^57^ The use of and impact of care from allied health assistants (eg, physiotherapy assistant or occupational therapy assistant) is also understudied but shows promise in terms of increased therapy time and patient satisfaction.^58^ Leveraging the volunteer sector may not be a suitable alternative for some patients with significant complex care needs, but could be considered for some patient populations. These areas of study require further research to determine promise in supporting various interventions including our co‐design work.

## 5 LIMITATIONS

Our sample was largely Caucasian‐ and English‐speaking whose experiences likely do not reflect participants from other backgrounds. Despite efforts, we were only able to engage one physician in the co‐design process, and given their critical role in designating ALC patients, incorporating their perspective in the future will be important. At each stage of research, the core issues for which we were co‐designing strategies were consistent, and the proposed components for a future intervention were agreed upon by participants, with any additions reflective of local needs. Overall, our findings were relevant to each of our study sites. It is expected that additional adaptations will be required at the local level and this will be realized in a future feasibility study. The strength of this work is that our team partnered with patients and caregivers at every step of the research and captured the experiences of care providers as well. By taking our findings to different jurisdictions and building upon them, we were able to continually refine our findings and ensure relevance to diverse stakeholders. Through co‐creation, we developed strategies of relevance to people with lived experience.

## 6 CONCLUSION AND NEXT STEPS

By working in partnership with a Patient and Caregiver Advisory Council, we were able to design and conduct a study that meaningfully engaged people with lived experience (including point of care providers) to design components of a future intervention that aims to make the experience of waiting from hospital more manageable, clear and dignified.

Future research by our team will entail a feasibility study to determine how (and the extent to which) the communication and care services recommended in this paper can be operationalized on clinical units. This will lead to future pilot studies and a pragmatic trial to assess outcomes.

## CONFLICT OF INTEREST

The authors declare no conflict of interest.

## Data Availability

Research data are not shared.
